# Comparison of Gut Microbiota of 96 Healthy Dogs by Individual Traits: Breed, Age, and Body Condition Score

**DOI:** 10.3390/ani11082432

**Published:** 2021-08-18

**Authors:** Inhwan You, Min Jung Kim

**Affiliations:** 1 Department of Research and Development, Mjbiogen Corp., 144 Gwangnaru-ro, Seongdong-gu, Seoul 14788, Korea; yih.mjbiogen@gmail.com; 2 College of Veterinary Medicine, Seoul National University, Seoul 08826, Korea

**Keywords:** gut microbiota, dog, companion animal, breed, age, body condition score, *Fusobacterium perfoetens*

## Abstract

**Simple Summary:**

The gut microbial ecosystem is affected by various factors such as lifestyle, environment, and disease. Although gut microbiota is closely related to host health, an understanding of the gut microbiota of dogs is still lacking. Therefore, we investigated gut microbial composition in healthy dogs and divided them into groups according to their breed, age, or body condition score. From our results, age is the most crucial factor driving the gut microbial community of dogs compared to breed and body condition score (especially *Fusobacterium perfoetens*, which was much more abundant in the older group). We have revealed that even in healthy dogs without any diseases, there are differences in gut microbiota depending on individual traits. These results can be used as a basis for improving the quality of life by managing dogs’ gut microbiota.

**Abstract:**

Since dogs are part of many peoples’ lives, research and industry related to their health and longevity are becoming a rising topic. Although gut microbiota (GM) is a key contributor to host health, limited information is available for canines. Therefore, this study characterized GM according to individual signatures (e.g., breed, age, and body condition score—BCS) of dogs living in the same environment. Fresh fecal samples from 96 healthy dogs were analyzed by sequencing the V3-V4 region of the 16S rRNA gene. The major microbial phyla were Firmicutes, Bacteroidetes, Fusobacteria, Proteobacteria, and Actinobacteria. In the comparison by breeds, relative abundance of Fusobacterium was significantly differed. Interestingly, *Fusobacterium perfoetens* abundance was positively correlated with age (*p* = 0.018), being significantly more enriched in the 6–10-year-old group (14.3%) than in the 0.5–1-year-old group (7.2%). Moreover, despite the healthy appearance of dogs in all age (0.5–10 years) and BCS (3–6) groups, the gut microbial environment may be disadvantageous in older dogs or in dogs with an abnormal BCS. These findings broaden our understanding of gut microbial ecology according to individual characteristics of dogs and may be used as a reference for providing customized-care to companion animals.

## 1. Introduction

Most microorganisms in the mammalian body reside in the intestinal tract, and numerous studies over the past decades have revealed a close relationship between gut microbiota (GM) and a healthy life. GM supports the maintenance of the intestinal mucus layer, secretes microbial compounds (e.g., bacteriocin and lactic acid) that suppress pathogens [1,2], and meditates host metabolic capabilities with bacterial metabolites [3,4,5]. A well-balanced and stabled GM greatly contributes to maintaining homeostasis and promptly responds to infections in dogs [6], and gut microbial dysbiosis in dogs is associated with several diseases such as metabolic disorders [7], inflammatory bowel disease [8,9], and arthritis [10]. Recent works based on molecular techniques have revealed that the most identified bacterial sequences in the canine gastrointestinal tract are from the following five phyla; Firmicutes, Fusobacteria, Bacteroidetes, Proteobacteria, and Actinobacteria. However, even in healthy host without diseases, GM is also affected by various factors, including birth mode, diet, stress, as well as their geographic location [11,12]. Therefore, we hypothesized that GM could be affected by dogs’ breed, age, or weight.

There are over 400 dog breeds, exhibiting a greater intraspecific phenotypic diversity than any other mammal. Heavy genetical modifications over time resulted in a unique phenotypic diversity influencing body and head shape, lifespan, and intelligence. Moreover, a link between the predisposition to certain gastrointestinal conditions and dog breeds has been established [13,14,15,16,17].

Age is one of the main factors inducing significant shifts in GM community with a decline in microbial diversity [18]. As the organism ages and goes through immune-senescence and inflammatory-aging, GM as well undergoes continual changes with diet, physical activity, and drug use. In elderly people, beneficial microbes populations such as *Akkermansia muciniphila*, which prevent leakage and subsequent induction of inflammation, are decreased [19,20]. In addition, dysfunction of gut-brain axis has been associated with neurodegenerative disorders [21,22]. In old dogs, a cognitive decline can be observed through human-like learning and memory deficits [23].

Many studies have compared the GM composition of overweight or obese individuals and have revealed several characteristics linked with these conditions, including the Firmicutes/Bacteroidetes ratio [24]. High abundance of Bacteroidetes in the gut microbiota has been associated with fecal concentration of short chain fatty acids (SCFAs) which interact with G-protein coupled receptors (GPCRs) [25]. GPCRs modulate various metabolic functions, including leptin secretion, which induces satiety and reduces food intake. However, in dogs, the differences in GM between obese and normal are still controversial [26].

For these reasons, dogs’ GM characterization according to individual characteristics, is imperative, in addition to general profiling. The present study compared the GM of 96 healthy dogs according to breed, age, and body condition score (BCS) based on *16S rRNA* sequences. All dogs examined in this study lived within the same environment (place, lifestyle, and owner) to reduce the influence of external factors.

## 2. Materials and Methods

### 2.1. Animals

A total of 96 healthy dogs (60 females and 36 males) of 9 different breeds (2 Greyhound, 6 Dachshund, 28 Maltese, 5 Bichon, 3 Yorkshire terrier, 5 Chihuahua, 6 Pomeranian, 34 Poodle, and 7 Bulldog) were enrolled. Dogs were fed with a commercial diet (30% crude protein, 17% crude fat, 6% crude fiber, 10% crude ash, 1% calcium, 0.6% phosphorus, and 12% moisture) and always had access to water. Each dog was housed in a single cage under the same environment by one owner, who is professional breeder (Gwangju, South Korea). Dogs were maintained routinely before and after experiment, and there was no additional treatment for this study. No dog presented a history of medication, neutralization, or diarrhea in the past four months. All experimental procedures in this study were approved by the Committee for Accreditation of Laboratory Animal Care and the Guideline for the Care and Use of Laboratory Animals of Seoul National University (approval number is SNU-200424-4).

### 2.2. Experimental Design

For the comparison by breed, dogs were divided into nine groups; Greyhound, Dachshund, Maltese, Bichon, Yorkshire Terrier, Chihuahua, Pomeranian, Poodle, and Bulldog. In the comparison by age, they were divided into three age groups; 0.5–1, 2–5, and 6–10 years old, corresponding to adolescence, adulthood, and old adults, respectively. For the comparison by obesity status, dogs were divided into three groups according to their body condition score (BCS). BCS was estimated by a veterinarian based on a 9-point scale, which is the clinical method of assessing body fat accumulation [27,28]. Three BCS groups consisted of BCS 3, BCS 4–5, and BCS 6–8 each corresponding to lean, normal/ideal, and overweight. Briefly, 16, 63, and 17 dogs were enrolled in 0.5–1-, 2–5-, and 6–10-year-old group, and 7, 84, and 5 dogs were enrolled in BCS 3, BCS 4–5, and BCS 6–8 group, respectively. The dogs’ GM were compared according to breed, age, and BCS based on *16S rRNA* sequences.

### 2.3. Sample Collection and DNA Extraction

Rectal swab samples from dogs were collected using N-SWAB TRANSPORT (Noble Bio, Hwaseong, Korea) and transported to the laboratory at 4 °C within 2 h. All samples were stored at −80 °C until further experiments. Genomic DNA was extracted using the DNeasy PowerSoil Kit (Qiagen, Hilden, Germany) following the manufacturer’s instructions and quantified using Quant-iT PicoGreen (Invitrogen, Waltham, MA, USA).

### 2.4. Library Construction and Sequencing

DNA libraries were constructed using Illumina 16S Metagenomic Sequencing Library Prep Guide to amplify the V3-V4 region. gDNA (2 ng) was amplified with 5× reaction buffer, 1 mM dNTP mix, 500 nM each of the universal F/R primers, and Herculase II fusion DNA polymerase (Agilent Technologies, Santa Clara, CA, USA). The amplification conditions were as follows: heat activation at 95 °C for 3 min; 25 cycles of 95 °C for 30 s, 55 °C for 30 s, and 72 °C for 30 s; and final extension at 72 °C for 5 min. The following universal primers with adapter overhang sequences (Illumina, San Diego, CA, USA) were used: V3-F, 5′-CGTCGGCAGCGTCAGATGTGTATAAGAGA CAGCCTACGGGNGGCWGCAG-3′, and V4-R, 5′-GTCTCGTGGGCTCGGAGATGTGTATAAGAG ACAGGACTACHVGGGTATCTAATCC-3′. The polymerase chain reaction (PCR) product was purified using AMPure beads (Agencourt Bioscience, Essex County, MA, USA). Following purification, the PCR product (2 μL) was amplified for final library construction using Nextera XT Indexed Primer. The cycling conditions and purification steps for this PCR were the same as above, except the use of 10 cycles. The final purified product was quantified using the qPCR Quantification Protocol Guide (KAPA Library Quantification Kit Illumina Platforms, Cape town, South Africa) and qualified using the Agilent D1000 ScreenTape System (Agilent Technologies, Waldbronn, Germany). Paired-end (2 × 300 bp) sequencing was performed by Macrogen (Seoul, Korea) using the MiSeq™ platform (Illumina, San Diego, CA, USA).

### 2.5. Gut Microbial Analysis

The MiSeq result was converted to FASTQ files based on Illumina index sequences. The adapter sequences were trimmed using FASTP [29], and overlapping regions were demultiplexed. Sufficiently short reads were extended by overlapping paired-end reads FLASH v1.2.11 [30]. After removing the < 400 or > 500 bp sequences, the remaining reads were clustered into operational taxonomic units (OTUs) with 97% sequence similarity using CD-HIT-OTU [31]. Taxonomic assignment was performed using BLASTN 2.4.0, with ≥ 85% query coverage and identity score [32]. The observed OTUs were used for microbial community analysis using QIIME 1.9 [33].

### 2.6. Statistical Analysis

Alpha diversity was evaluated by calculating Observed OTUs, Chao1, the Shannon index, and the Inversed Simpson index using QIIME 1.9 to measure within groups microbial diversity. To compare of microbial composition between groups, beta diversity was estimated based on unweighted and weighted UniFrac distances and visualized using principal coordinate analysis (PCoA). All other analyses and visualizations were performed with R software version 3.0.1. and the boxplot package. For all statistical analyses, *p* < 0.05 was statistically significant. In the comparison of microbial diversity index and relative abundance, the Kruskal–Wallis test or Wilcoxon rank sum test were used depending on the number of compared groups to determine significantly (*p* < 0.05) varied among groups [34,35]. Linear discriminant analysis (LDA) effect size (LEfSe) [36] was used to compare the abundance distribution among taxa (*p* < 0.05, with LDA score > 2.0 or 3.0).

## 3. Results

### 3.1. Overall GM of Healthy Dogs

A total of 3,553,326 high-quality reads were obtained from the 96 healthy dogs enrolled. We analyzed fecal samples from dogs of nine breeds, aged 0.5 to 10 years and with a BCS of 3 to 8. A total of 1254 bacterial OTUs classified into 14 phyla, 27 classes, 56 orders, 106 families, 286 genera, and 533 species were obtained.

**Figure 1:**
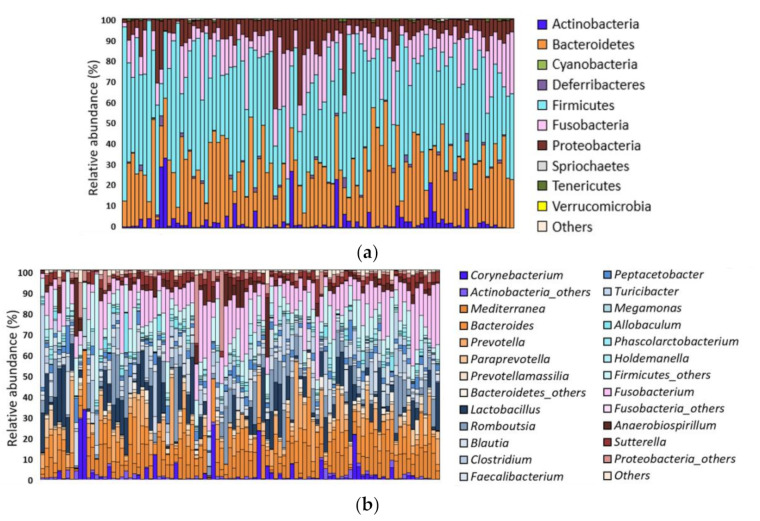
Overall gut microbial composition of 96 healthy dogs. Relative abundances of (**a**) phyla (≥0.01% of the total sequences) and (**b**) genera (≥1% of the total sequences) from the five major phyla.

The following five phyla were predominant (≥1% of total sequences), accounting for over 98% of total *16S rRNA* sequences in the dog GM: Firmicutes (44.8%), Bacteroidetes (27.7%), Fusobacteria (14.2%), Proteobacteria (8.8%), and Actinobacteria (3.4% of total average abundance) (Figure 1). At the genus and species level, respectively, 20 and 28 taxa were predominant (≥1% of the total sequences). The predominant species included *Fusobacterium perfoetens* (0–59%), *Mediterranea massiliensis* (0–22%), *Prevotella copri* (0–38%), and *Romboutsia timonensis* (0–36%) (Table S1).

### 3.2. Breed

**Figure 2:**
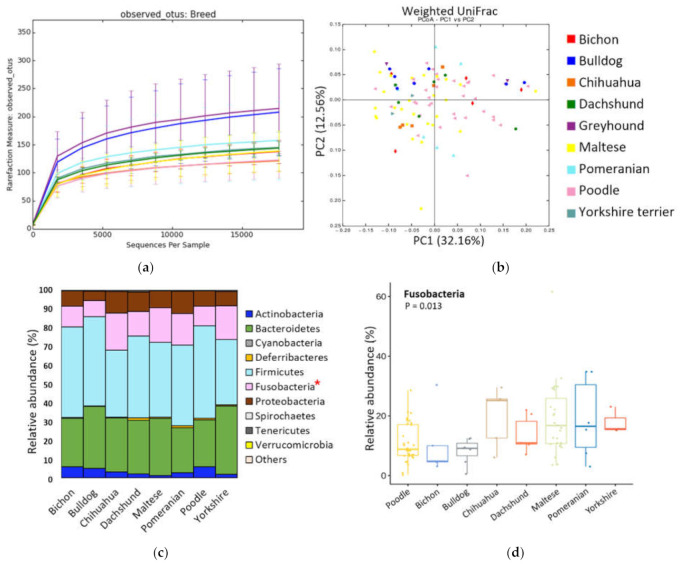
Alpha-, beta diversity, and relative abundances at the phylum level according to breed. (**a**) Microbial richness was measured based on observed OTUs. (**b**) PCoA was performed based on weighted UniFrac distances. (**c**) Relative abundance at the phylum level. (**d**) Box plot of the abundance of Fusobacteria that showed significant differences among breeds. * Indicates that significantly different bacteria group.

Among 9 breeds Greyhound breed was excluded from the comparison as there were only two individuals. Microbial richness (observed OTUs) was significantly higher in Bulldog (*p* = 0.04), but microbial evenness (Shannon index) did not differ by breed (*p* = 0.17). Samples were not clustered by breed in PCoA based on unweighted (Figure S1) and weighted UniFrac distances (Figure 2b).

Phylum Fusobacteria was significantly differed according to breed (*p* = 0.01) (Figure 2d). Twenty-one genus and twenty-six species showed significant differences (*p* < 0.05), especially three species (*Fusobacterium perfoetens*, *Romboutsia timonensis*, and *Sutterella stercoricanis*) among them had an abundance of over 5.0% (Table S2).

According to LEfSe results performed between the Maltese and Poodle groups, which had the highest number of samples, showed that the abundance distribution of taxa was clearly differed between the two groups (*p* < 0.05 and LDA > 3.0) (Figure S2). In Malteses, Fusobacteria were abundant, whereas in Poodles, Firmicutes and Actinobacteria were abundant. The results for all taxonomic levels are presented as an LDA bar graph. Moreover, there were significant differences in the abundance of four phyla and sixteen species between these two breeds (*p* < 0.05). The number of shared and unique OTUs between the two breeds were visualized via Venn diagram (Figure S7).

### 3.3. Age

**Figure 3:**
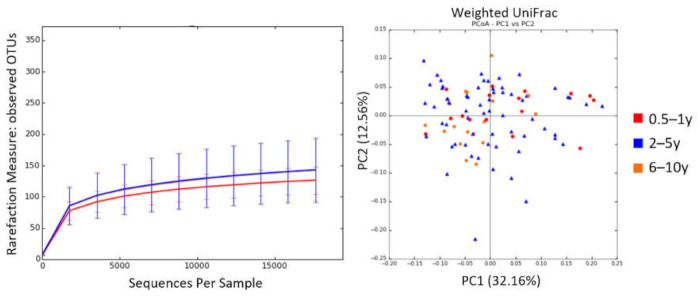
Alpha and beta diversity according to dog age group. Microbial richness was measured based on observed OTUs (**left**). PCoA was performed based on weighted UniFrac distances (**right**).

No age-related differences were found in alpha and beta diversity (Figure 3 and Figure S3), but several bacterial groups showed different abundances.

**Figure 4:**
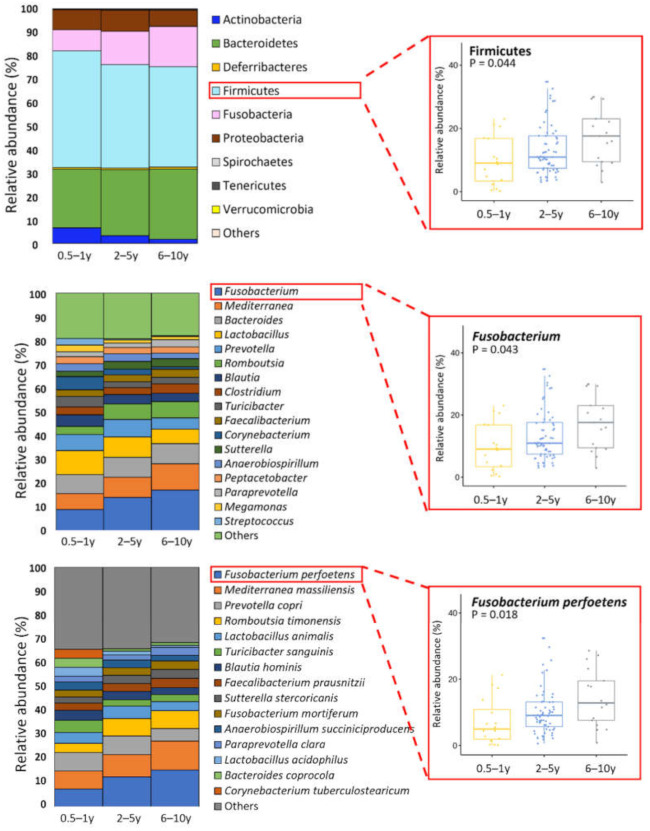
Relative abundances of bacteria at the phylum, genus, and species levels by the age of dogs. Major bacterial groups were selected for chart analysis (phylum ≥ 0.01%, genus ≥ 2%, and species ≥ 3% of total abundance). * Indicates that significantly different bacteria group.

**Figure 5:**
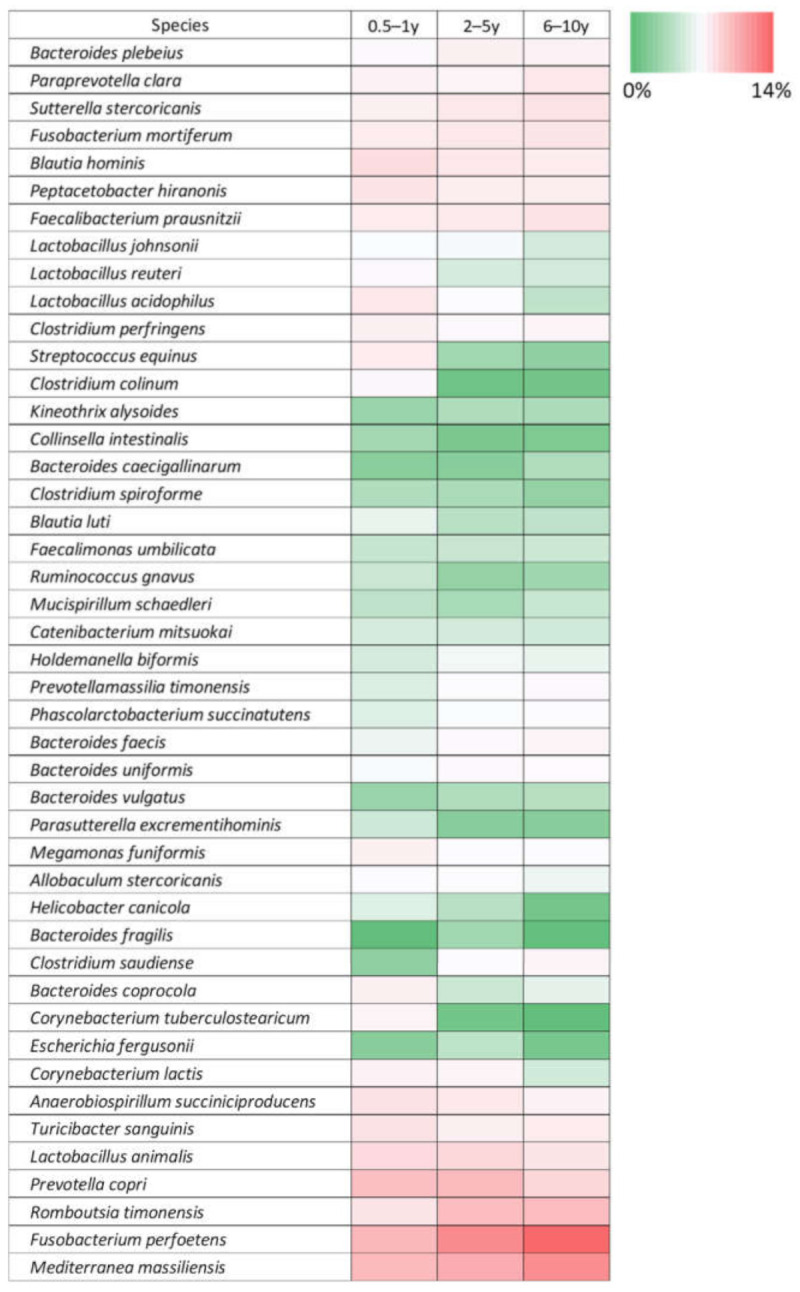
Heatmap of gut microbiota at the species level between different age groups. Color scale based on relative abundance of operational taxonomic unit (OTU). Forty-five core species were included in this analysis.

Phylum Fusobacteria (*p* = 0.05), genus *Jeotgalicoccus* (*p* = 0.02), *Faecalibaculum* (*p* = 0.03), and *Fusobacterium* (*p* = 0.04) were significant differed. Seven species showed age-dependent differences (*p* < 0.05) (Table S3). Specifically, *Fusobacterium perfoetens* was the most significantly affected species by dog age (Figure 4). Figure 5 indicates the distribution of forty-five core species according to age. LEfSe analysis revealed similar trends (*p* < 0.05, LDA score > 3.0) (Figure S4).

According to Wilcoxon rank sum test, respectively 12, 4, and 8 species significantly differed between the 0.5–1- and 2–5-year-old groups, between the 2–5- and 6–10-year-old groups, and between the 0.5–1- and 6–10-year-old groups. *F. perfoetens* was approximately two times more abundant in the 6–10-year-old group than in the 0.5–1-year-old group (14.3% vs. 7.2%).

### 3.4. BCS

**Figure 6:**
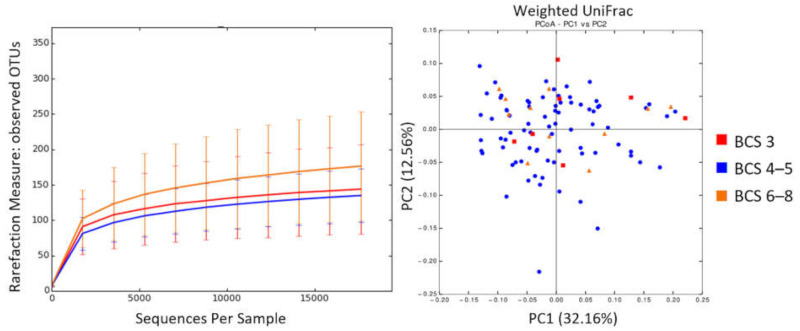
Alpha and beta diversity according to dog BCS groups. Microbial richness was measured based on observed OTUs (**left**). PCoA was performed based on weighted UniFrac distances (**right**).

**Figure 7:**
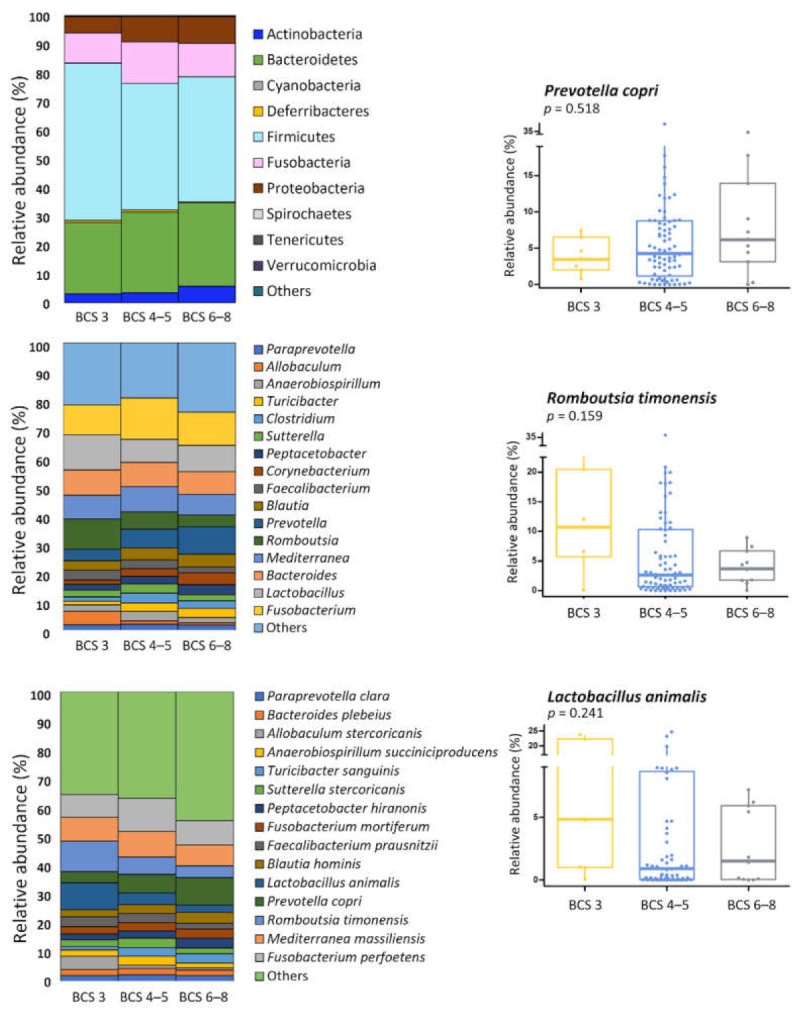
Gut microbial composition and box plots in different body condition score (BCS) groups. Major bacterial groups at the phylum (≥0.01%), genus (≥2%), and species (≥2%) level. Box plot represents no significant difference (*p* > 0.05) but shows bacterial groups that tend to increase or decrease with BCS. * Indicates that significantly different bacteria group.

**Figure 8:**
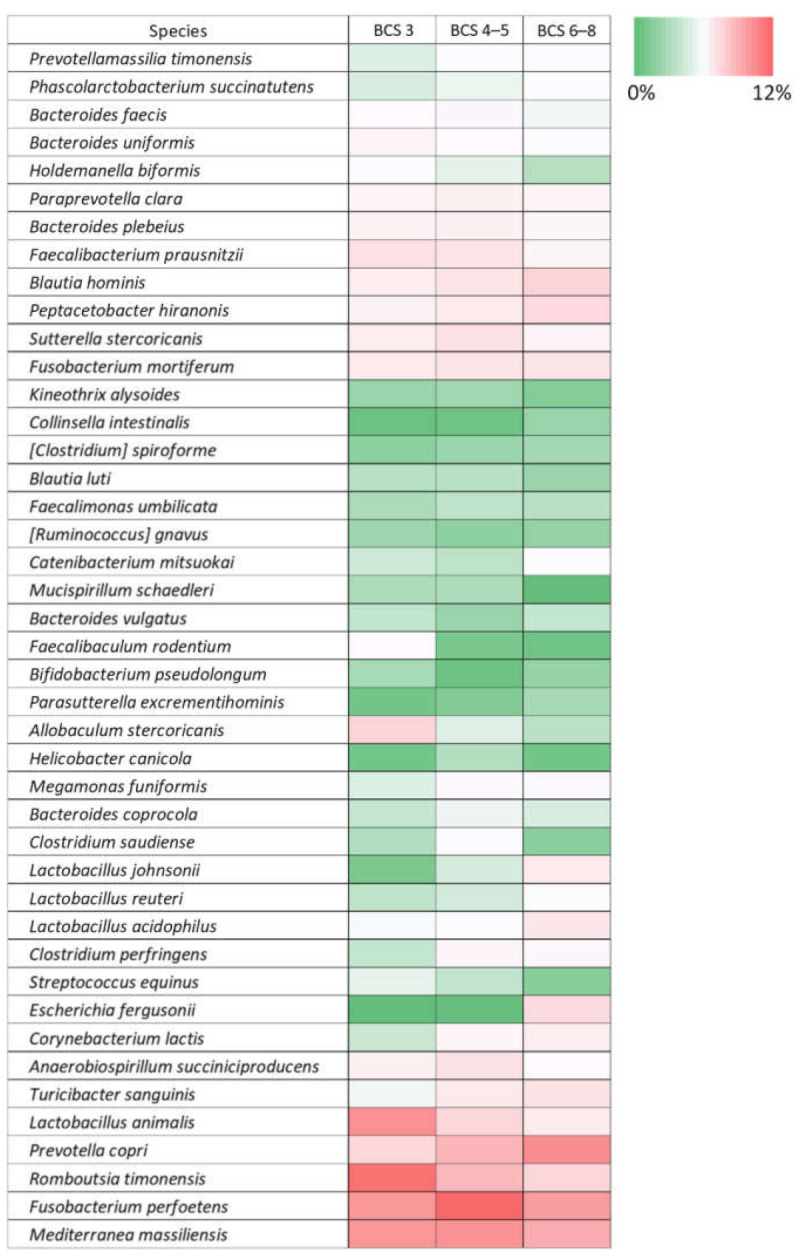
Heatmap of gut microbiota at the species level between different body condition score (BCS) groups. Color scale based on relative abundance of operational taxonomic unit (OTU). Forty-three core species were included in this analysis.

There were no significantly differences in alpha and beta diversity according to BCS (Figure 6 and Figure S5). At the phylum level, Actinobacteria was significantly more abundant in the overweight group than in the other groups (Figure 7). At the genus and species level, several taxa showed significant differences among the three BCS groups, but their average abundance was < 0.1% of the total *16S rRNA* sequences (Table S4). Although there was no statistical significance, the abundance of *Romboutsia timonensis* and *Lactobacillus animalis* tended to decrease with increasing BCS, and *Prevotella copri* increase (Figure 7). Forty-three core species were obtained between different BCS groups, and the distribution was showed in Figure 8.

When comparing the two of the three groups, the difference at the phylum level existed only in the comparison of the normal vs. overweight group. The overweight group showed higher Actinobaceria and Deferribacteres than the normal group. Also, six species (*Enterococcus cecorum, Peptostreptococcus russellii, Bacteroides fragilis, Bacteroides massiliensis, Bacteroides thetaiotaomicron*, and *Corynebacterium lactis*) showed higher levels in the overweight group, whereas three species (*Anaerobiospirillum succiniciproducens*, *Sutterella stercoricanis*, and *Mucispirillum schaedleri*) had lower. In the comparison of lean vs. normal group, the lean group showed higher abundance of *Streptococcus equinus*, *Peptostreptococcus russellii* and *Beduini massiliensis*. In the comparison of lean vs. overweight group, *Anaerobiospirillum succiniciproducens, Beduini massiliensis, Rouboutsia timonensis*, and *Streptococcus equins* showed higher levels in the lean group (data not shown).

In LEfSe analysis of the three groups, the overweight group was enriched in *Peptostreptococcus russellii*, *Schaalia cardiffensis*, and *Enterococcus cocerum*; the lean group in *Fenollaria*; and the normal group in *Cellulosilyticum ruminicola* (Figure S6).

## 4. Discussion

In humans, gut microbial community composition greatly varies according to individual traits and environmental condition [12,37,38]. Similarly, in dogs, gut microbial composition may vary according to breed, age, or other conditions. Here, we characterized the GM of 96 healthy dogs, who belonged to nine different breeds, aged between 0.5 to 10 years, with a BCS ranging from 3 to 8, and living under the same environmental conditions. The predominant phyla in this study were consistent with previous reports in canines: Firmicutes, Bacteroidetes, Fusobacteria, Proteobacteria, and Actinobacteria [37,38,39]. Except for Greyhounds, Bulldogs showed a higher number of obtained OTUs than the other breeds. Bulldogs are classified as medium-sized dogs, and all other breeds are small-sized dogs, but no study has yet reported this association. When Poodle and Maltese groups result were compared, there was a clear difference in microbial composition between the two breeds (Figure S2). Furthermore, similar to our study, a previous study comparing three breeds (Maltese, Poodle, and Miniature Schnauzer) showed phylum Fusobacterium was differed by breed, and the levels of Firmicutes were significantly lower in Maltese dogs [40]. These results suggest that there may be differences in the GM composition depending on the dog breeds. Further research with a bigger sample size per breed, would allow for a more precise comparison of GM by breed and body size in dogs.

Age groups were divided according to human years corresponding to adolescence, adulthood, and old adults [41]. In humans, gut microbial diversity decreases with aging. However, we found no such trend in the present study. Meanwhile, like in humans, we observed drastic age-related changes in the gut microbial composition from 0.5–1 years of age onward in dogs. Adolescence a period to occur physical and emotional changes with rapid growth. Many human studies have suggested that the gut microbial composition in early life affects the immune system and health in the future [42,43]. Our results suggest that the management of GM during the growing period is also important in dogs. In addition, bacteria that are more abundant in older adults may be age-dependent.

*F. perfoetens* abundance was positively correlated with age. Consistent with our results, Xu et al. reported that *F. perfoetens* was significantly more abundant in dogs aged 60–156 months than in younger ones (age < 8 months) [44]. *Fusobacterium* is a commensal bacterium living in healthy humans and dogs’ guts. However, several studies have reported that *Fusobacterium* was enriched in diverse models of intestinal diseases, including colorectal cancers and tumors [45,46,47]. We also found that this species may be associated with intestinal diseases. In a preliminary study, we compared samples from bloody stool and normal feces of the same individual. However, this result was obtained from one individual only, and further evidence are still needed, *F. perfoetens* was the bacterium with the greatest difference between bloody and normal stool sample. *F. perfoetens* was the most dominant bacterium in the bloody stool sample (46.9%), with low abundance in the normal stool sample (19.5%) (Data not shown). Although *Fusobacterium* is a commensal turned pathogen, previous studies have only focused on *F. nucleatum*. Our observations suggest that *F. perfoetens* is an aging-related or opportunistic pathogen, although in-depth studies of *F. perfoetens* including metabolic pathway, metabolites, and their associations with older dogs (more than 10 years-old) are needed.

In humans and mice, obesity is associated with decreased microbial diversity and an increased Firmicutes/Bacteroidetes (F/B) ratio [24,48,49,50]. However, no differences in the alpha and beta diversity or F/B ratio according to BCS were obtained in this study, similar to a previous study [26]. Actinobacteria was significantly enriched in the overweight group compared to the others. A higher proportion of Actinobacteria has been observed in obese individuals [48,51]. Actinobacteria is a saccharolytic bacteria, whose main metabolic end products are SCFAs [52], such as *Bacteroidetes*, and the fecal SCFA content was higher in obese individuals than in lean ones [53]. SCFAs production increases energy harvesting from diet and interferes with energy homoeostasis of the host [54]. Thus, Actinobacteria may be associated with obesity in dogs, as reported by many studies in humans. Some bacterial groups associated with disease or obesity showed higher levels in the overweight group than in the normal group. Among them, Deferribactere is related to intestinal iron metabolism, and higher levels of Deferribacteres increases iron metabolism. Abnormal iron metabolism could be promotes tumor growth or risk of cancer [55]. *Bacteroides fragilis, Bacteroides massiliensis*, and *Bacteroides thetaiotaomicron* are a group of Bacteroides, and are known to be positively correlated with obesity [50,56], showed more abundant in the overweight group.

At the species level, *P. russellii* showed a lower abundance in the normal group than in the other groups. This species reduces susceptibility to intestinal injury via the action of a cluster of genes encoding phenylacetate dehydratase [57,58]. Therefore, an ideal BCS (4–5) would be more conducive to the response and recovery of intestinal damage than BCS associated with under- or overweight conditions. *P. copri* showed a tendency to increase as BCS increased, and *L. animalis* showed the opposite trend. The beneficial effects of *P. copri* may be diet-dependent [59,60,61], but there was no dietary factor as all dogs in the present study received the same diet. Since *P. copri* is both beneficial and harmful, its effects on and reasons for its abundance in obese dogs should be examined. Oral administration of *L. animalis* to dogs increased fecal *Lactobacillus* abundance [62,63,64], while in dogs < 24 months of age, the abundance of *L. animalis* was negatively correlated with the level of TNF-α, which is related to obesity [44]. Therefore, we suggested that even in healthy individuals, a higher BCS may suppress some beneficial bacteria and promote opportunistic pathogens.

## 5. Conclusions

Our findings contribute to the understanding of canine GM which has not yet been fully refined and suggest variability according to individual traits. In healthy dogs, *Fusobacterium* suggests the potential to be an indicator of dog breeds characteristic. Additionally, regulating *F. perfoetens*, which is positively correlated with older dogs, might help promoting a healthy aging process in dogs. Finally, maintaining an ideal BCS not only prevents disease but also inhibits opportunistic pathogens. These findings can serve as a reference for ensuring companion animals’ well-being.

## Data Availability

Data will be available on request from the first author.

## Supplementary Materials

The following are available online at https://www.mdpi.com/article/10.3390/ani11082432/s1. Figure S1: Alpha and beta diversity according to dog breed; Figure S2: Linear discriminant analysis effect size (LEfSe) between Maltese and Poodle; Figure S3: Alpha and beta diversity according to dog age; Figure S4: Linear discriminant analysis effect size (LEfSe) according to age; Figure S5: Alpha and beta diversity according to body condition scores (BCS) of dogs; Figure S6: (LEfSe) according to BCS; Figure S7: Venn diagrams indicating the number of unique and shared operational taxonomic unit (OTUs); Table S1: Relative abundance of gut microbiota in 96 healthy dogs; Table S2: Relative abundance at the phylum and species level according to breed; Table S3: Relative abundance at the phylum and species level according to age; Table S4: Relative abundance at the phylum and species level according to BCS.
