# Supplementary material for: Comparison of Gut Microbiota of 96 Healthy Dogs by Individual Traits: Breed, Age, and Body Condition Score
Source: Animals (Basel). 2021 Aug 18;11(8):2432. doi: 10.3390/ani11082432 (PMC8388711; doi:10.3390/ani11082432)

Supplementary Table S1. Relative abundance of gut microbiota in 96 healthy dogs. Major bacterial groups (≥ 0.1%) are listed.

	Mean	SEM
**Phylum**		
Actinobacteria	1.3	0.6
Bacteroidetes	27.9	1.2
Firmicutes	41.7	1.7
Fusobacteria	11.0	1.0
Proteobacteria	6.5	0.8
**Genus**		
Fusobacterium	11.0	1.0
Mediterranea	7.8	0.5
Lactobacillus	3.5	0.9
Bacteroides	7.5	0.5
Prevotella	4.6	0.8
Romboutsia	3.1	0.8
Blautia	3.3	0.3
Clostridium	1.3	0.5
Anaerobiospirillum	1.7	0.5
Sutterella	2.5	0.2
Faecalibacterium	2.1	0.3
Peptacetobacter	2.2	0.2
Turicibacter	0.8	0.5
Corynebacterium	0.3	0.6
Paraprevotella	1.8	0.2
Megamonas	0.9	0.2
Prevotellamassilia	1.0	0.1
Allobaculum	0.7	0.2
Phascolarctobacterium	1.0	0.1
Holdemanella	0.7	0.1
**Species**		
Fusobacterium perfoetens	9.0	0.9
Mediterranea massiliensis	7.8	0.5
Prevotella copri	4.6	0.7
Romboutsia timonensis	3.1	0.8
Lactobacillus animalis	1.0	0.6
Blautia hominis	2.5	0.3
Sutterella stercoricanis	2.4	0.2
Fusobacterium mortiferum	2.4	0.3

Anaerobiospirillum succiniciproducens	1.5	0.5
Faecalibacterium prausnitzii	2.1	0.3
Peptacetobacter hiranonis	2.2	0.2
Turicibacter sanguinis	0.8	0.5
Bacteroides plebeius	1.8	0.2
Paraprevotella clara	1.8	0.2
Clostridium perfringens	0.2	0.4
Corynebacterium lactis	0.1	0.5
Bacteroides faecis	1.4	0.1
Lactobacillus acidophilus	0.1	0.3
Megamonas funiformis	0.9	0.2
Bacteroides uniformis	0.9	0.2
Prevotellamassilia timonensis	1.0	0.1
Allobaculum stercoricanis	0.7	0.2
Clostridium saudiense	0.4	0.3
Phascolarctobacterium succinatutens	1.0	0.1
Bacteroides coprocola	0.8	0.3
Lactobacillus johnsonii	0.1	0.3
Holdemanella biformis	0.7	0.1
Lactobacillus reuteri	0.1	0.2

Supplementary Figure S1: Alpha and beta diversity according to dog breed. (a) Microbial richness and evenness were measured based on observed OTUs and Shannon index, respectively. (b) PCoA was performed based on un-weighted and weighted UniFrac distances. (c) List of alpha diversity indices between Poodle and Maltese.

(a)

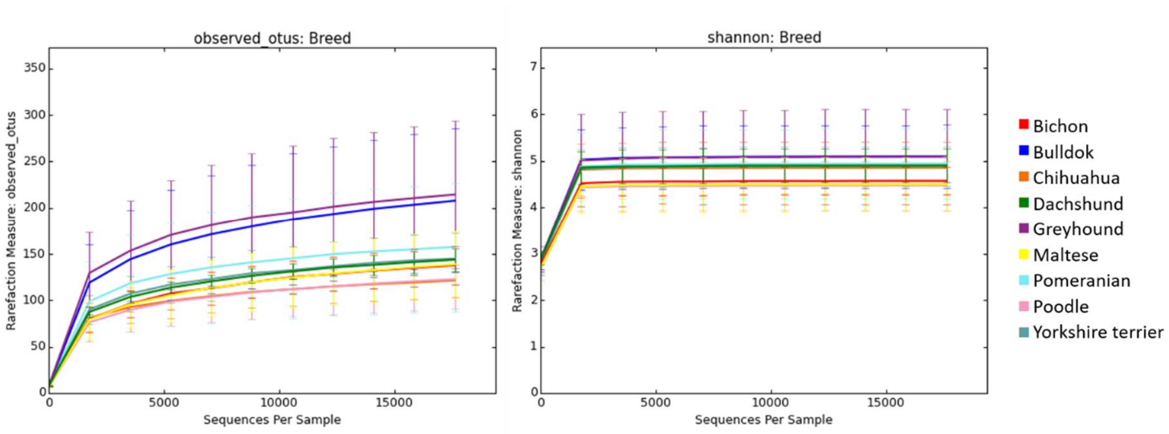

(b)

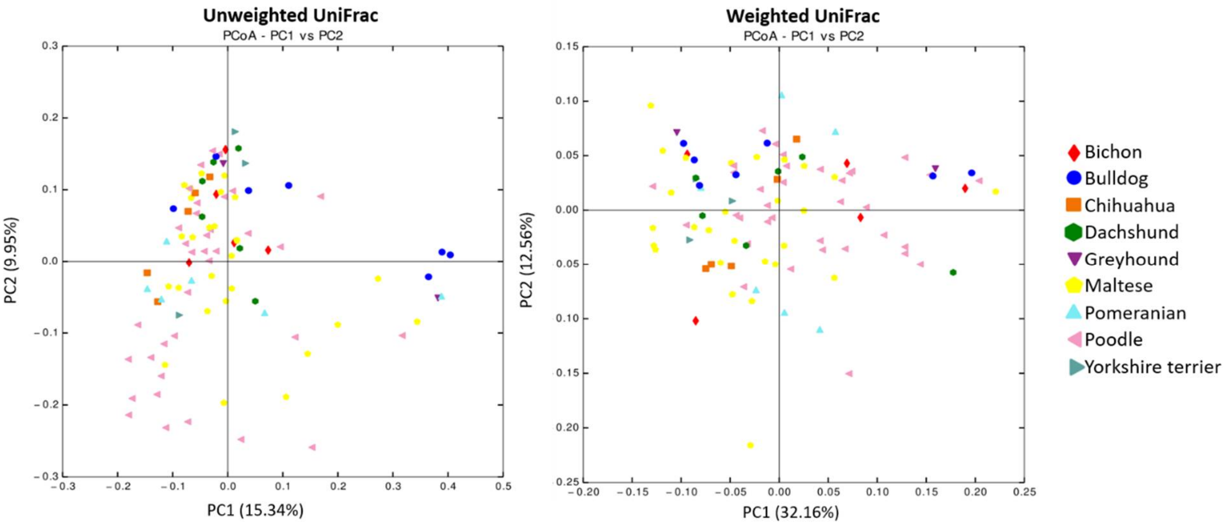

(c)

Diversity Index	Poodle		Maltese		95% Confidence Interval		p-value	FDR
	Mean	SEM	Mean	SEM	Lower	Upper		
Chao1	149.6	7.3	177.1	9.1	-48.9	-0.8	4.5E-02*	1.4E-01
Inverse Simpson	0.9	0.0	0.9	0.0	0.0	0.0	4.4E-01	5.8E-01
Shannon	4.5	0.1	4.5	0.1	-0.3	0.2	7.0E-01	7.0E-01
OTUs	134.5	6.2	157.2	7.5	-39.0	2.0	6.8E-02	1.4E-01

Supplementary Table S2. Relative abundance at the phylum and species level according to breed. Kruskal–Wallis test was used, and data are shown as the mean ± SEM. Major or significantly different bacterial groups (abundance ≥ 0.1% or $P < 0.05$) are shown. Asterisks indicate $P < 0.05$.

	**Bichon**	**Bulldog**	**Chihuahua**	**Dachshund**	**Maltese**	**Pome-ranian**	**Poodle**	**Yorkshire**	**p-value**	**FDR**
**Phylum**										
Fusobacteria	10.6±5.1	8.2±1.7	19.9±4.5	13.7±2.5	19±2.4	18.9±5.5	11.0±1.1	18.0±2.6	1.31E-02*	1.31E-02*
Proteobacteria	8.3±3.1	5.1±1.4	11.6±2.3	11.2±3.9	9.3±1.4	12.0±6.2	8.3±1.2	7.7±1.2	1.32E-01	1.85E-01
Actinobacteria	5.9±4.1	5.0±1.3	2.9±1.3	2.1±0.7	1.3±0.3	2.5±1.4	5.3±1.6	1.8±0.4	8.87E-02	1.85E-01
Firmicutes	49.3±10.5	49.3±8.6	35.6±5	43.3±3.3	39.1±2.8	41.3±5.3	49.8±2.8	34.9±1.9	1.20E-01	1.85E-01
Tenericutes	0.3±0.2	0.3±0.2	0.6±0.3	0.8±0.2	0.4±0.1	0.3±0.2	0.4±0.1	0.6±0.3	1.21E-01	1.85E-01
Bacteroidetes	25.5±7.6	31.9±6.1	29.2±0.6	27.9±6	30.6±2.5	23.8±4.4	24.6±1.9	36.2±3.6	2.12E-01	2.47E-01
Deferribacteres	0.5±0.4	0.2±0.1	0.4±0.1	1.1±0.6	0.6±0.2	1.1±0.6	0.8±0.3	0.8±0.4	3.19E-01	3.19E-01
**Species**										
Prevotella stercorea	0.2±0.2	0.1±0.1	0.0±0.0	0.1±0.1	0.6±0.4	0.0±0.0	0.1±0.1	0.0±0.0	4.57E-06*	1.19E-04*
Turicibacter sanguinis	5.4±1.8	2.8±0.9	0.3±0.1	0.4±0.3	2.0±0.6	0.2±0.2	3.3±0.8	0.4±0.3	7.09E-04*	9.22E-03*
Bifidobacterium longum	0.1±0.1	0.1±0.1	0.1±0.1	0.1±0.1	0.1±0.1	0.1±0.1	0.1±0.1	0.0±0.0	2.75E-03*	1.92E-02*
Sutterella stercoricanis	5.1±2.1	1.7±0.6	3.2±0.6	3.0±0.9	4.5±0.6	2.5±0.8	2.2±0.2	3.6±0.7	2.95E-03*	1.92E-02*
Fusobacterium perfoetens	7.1±4.1	5.5±1.3	16.5±4.5	11.0±2.4	15.9±2.4	16.0±5.5	7.9±0.8	14±2.1	6.01E-03*	2.30E-02*
Lawsonella clevelandensis	0.0±0.0	0.0±0.0	0.0±0.0	0.6±0.6	0.1±0.1	0.1±0.1	0.1±0.1	0.1±0.1	7.09E-03*	2.30E-02*
Helicobacter canicola	0.1±0.1	0.3±0.2	0.5±0.3	0.6±0.4	0.2±0.1	4.0±3.7	0.8±0.3	0.1±0.1	7.34E-03*	2.30E-02*
Corynebacterium maris	0.1±0.1	0.5±0.4	0.1±0.1	0.1±0.1	0.1±0.1	0.0±0.0	0.1±0.1	0.1±0.1	7.43E-03*	2.30E-02*
Caproiciproducens galactitolivorans	0.1±0.1	0.1±0.1	0.1±0.1	0.2±0.2	0.1±0.1	0.1±0.1	0.1±0.1	0.1±0.1	8.59E-03*	2.30E-02*
Butyricicoccus pullicaecorum	0.2±0.2	0.1±0.1	0.6±0.4	1.1±0.7	0.2±0.1	0.8±0.3	0.4±0.1	0.6±0.4	8.87E-03*	2.30E-02*

Porphyromonas cangingivalis	0.4±0.3	0.6±0.5	0.1±0.0	0.1±0.1	0.2±0.2	0.2±0.2	0.1±0.1	1.1±0.9	9.75E-03*	2.30E-02*
Helicobacter canis	0.1±0.1	0.1±0.1	0.3±0.3	0.1±0.1	0.1±0.1	0.4±0.4	0.1±0.1	0.1±0.1	1.22E-02*	2.64E-02*
Faecalitalea cylindroides	0.1±0.1	0.1±0.1	0.2±0.1	0.1±0.1	0.1±0.1	0.1±0.1	0.1±0.1	0.1±0.1	1.35E-02*	2.69E-02*
Odoribacter splanchnicus	0.1±0.1	0.1±0.1	0.1±0.1	0.1±0.1	0.1±0.1	0.1±0.1	0.1±0.1	0.1±0.1	1.75E-02*	3.04E-02*
Hungateiclostridium straminisolvens	0.1±0.1	0.1±0.1	0.5±0.5	0.1±0.1	0.1±0.1	0.1±0.1	0.1±0.1	0.1±0.1	1.76E-02*	3.04E-02*
Romboutsia timonensis	8.9±2.8	3.9±0.8	2.4±2.3	3.4±1.9	4.9±1.1	1.0±0.5	10.1±1.7	2.6±1.6	2.16E-02*	3.44E-02*
Streptococcus canis	0.1±0.1	0.1±0.1	0.1±0.1	0.1±0.1	0.1±0.1	0.1±0.1	0.1±0.1	0.4±0.4	2.29E-02*	3.44E-02*
Parasutterella excrementihominis	0.5±0.3	1.1±0.6	0.2±0.1	0.1±0.1	0.7±0.4	0.3±0.2	0.2±0.2	0.7±0.6	2.38E-02*	3.44E-02*
Peptococcus simiae	0.3±0.1	0.5±0.2	0.4±0.1	0.3±0.1	0.2±0.1	0.4±0.1	0.2±0.1	0.3±0.2	2.60E-02*	3.50E-02*
Intestinimonas butyriciproducens	0.0±0.0	0.2±0.1	0.0±0.0	0.1±0.1	0.1±0.1	0.2±0.2	0.1±0.1	0.0±0.0	2.73E-02*	3.50E-02*
Enterococcus cecorum	0.3±0.2	0.0±0.0	0.3±0.2	0.0±0.0	0.1±0.0	0.1±0.1	0.1±0.1	0.0±0.0	2.95E-02*	3.50E-02*
Corynebacterium ammoniagenes	0.2±0.2	0.5±0.4	0.0±0.0	0.1±0.1	0.1±0.1	0.0±0.0	0.2±0.1	0.4±0.3	2.96E-02*	3.50E-02*
Alistipes onderdonkii	0.1±0.1	0.1±0.1	0.0±0.0	0.1±0.1	0.1±0.1	0.1±0.1	0.1±0.1	0.0±0.0	3.44E-02*	3.89E-02*
Faecalibaculum rodentium	1.4±0.9	0.4±0.2	0.5±0.3	0.2±0.1	0.6±0.4	0.6±0.6	0.2±0.1	0.3±0.3	3.79E-02*	4.05E-02*
Coprococcus eutactus	0.0±0.0	0.1±0.1	0.0±0.0	0.0±0.0	0.1±0.1	0.1±0.1	0.1±0.1	0.0±0.0	3.96E-02*	4.05E-02*
Spiroplasma mirum	0.0±0.0	0.0±0.0	0.0±0.0	0.1±0.1	0.0±0.0	0.0±0.0	0.0±0.0	0.0±0.0	4.05E-02*	4.05E-02*

Supplementary Figure S2: Linear discriminant analysis effect size (LEfSe) between Maltese and Poodle. Cladogram showing different abundant taxa between the two breeds ($P < 0.05$, LDA scores ≥ 3) and bar graph showing LDA scores.

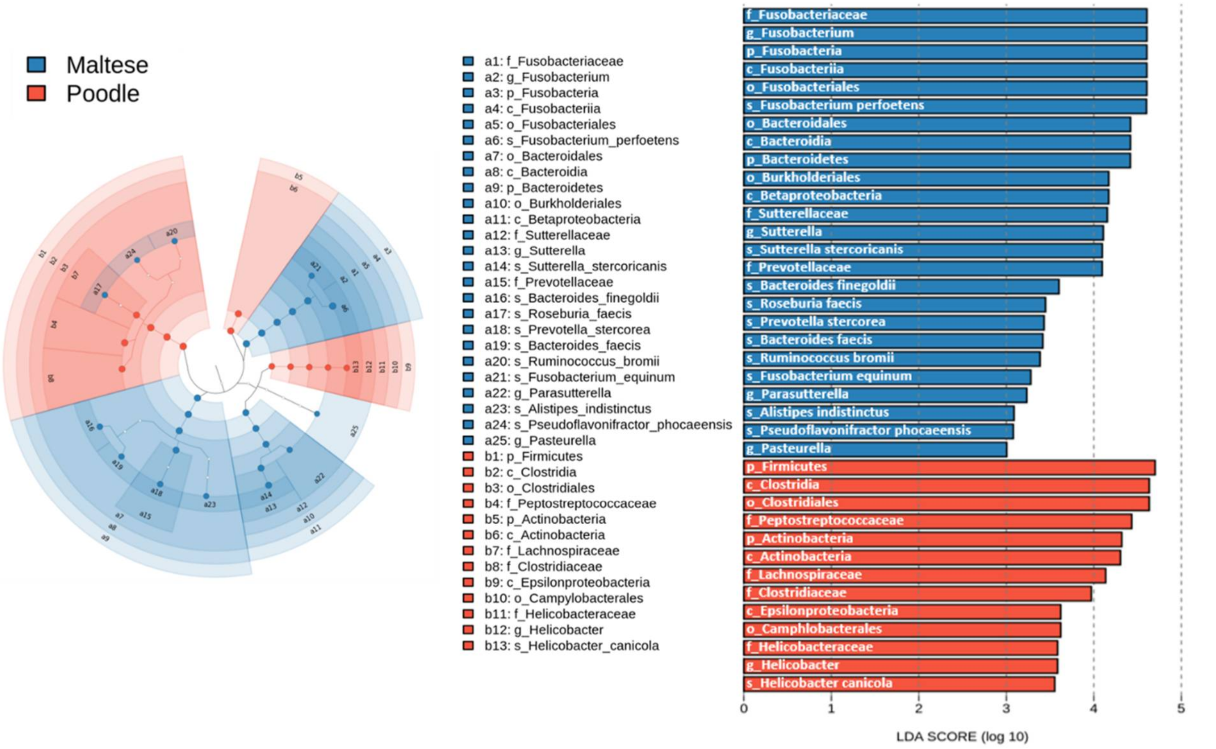

Supplementary Figure S3: Alpha and beta diversity according to dog age. (a) Microbial richness and evenness were measured based on observed OTUs and Shannon index, respectively. List of alpha diversity indices. (b) PCoA was performed based on un-weighted and weighted UniFrac distances.

(a)

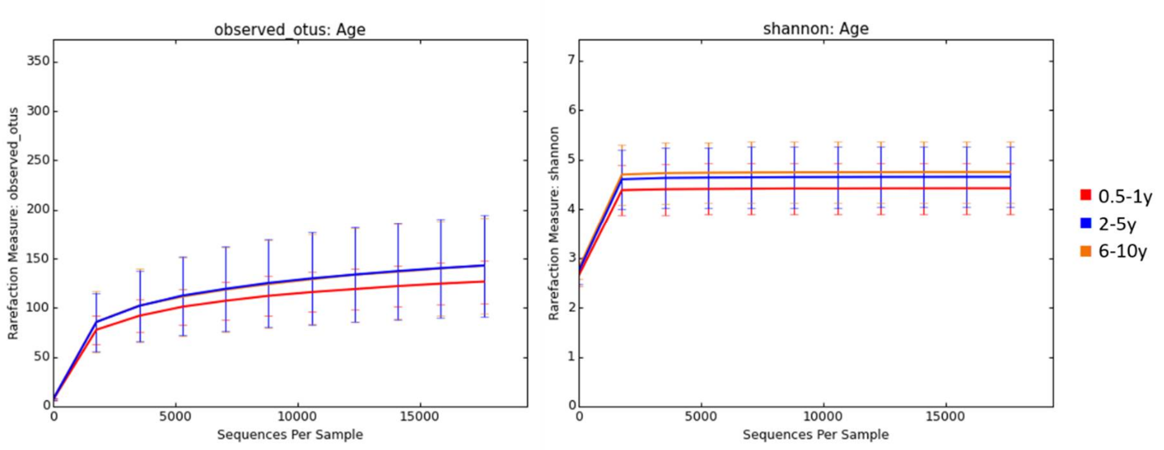

Diversity Index	0.5-1y		2-5y		6-10y		p-value	FDR
	Mean	SEM	Mean	SEM	Mean	SEM		
Chao1	150.1	7.0	176.2	8.4	181.7	14.4	3.9E-01	7.8E-01
Inverse Simpson	0.9	0.0	0.9	0.0	0.9	0.0	2.5E-01	7.8E-01
Shannon	4.4	0.1	4.7	0.1	4.8	0.2	5.9E-01	7.9E-01
OTUs	140.1	6.0	158.9	7.6	160.5	13.5	7.9E-01	7.9E-01

(b)

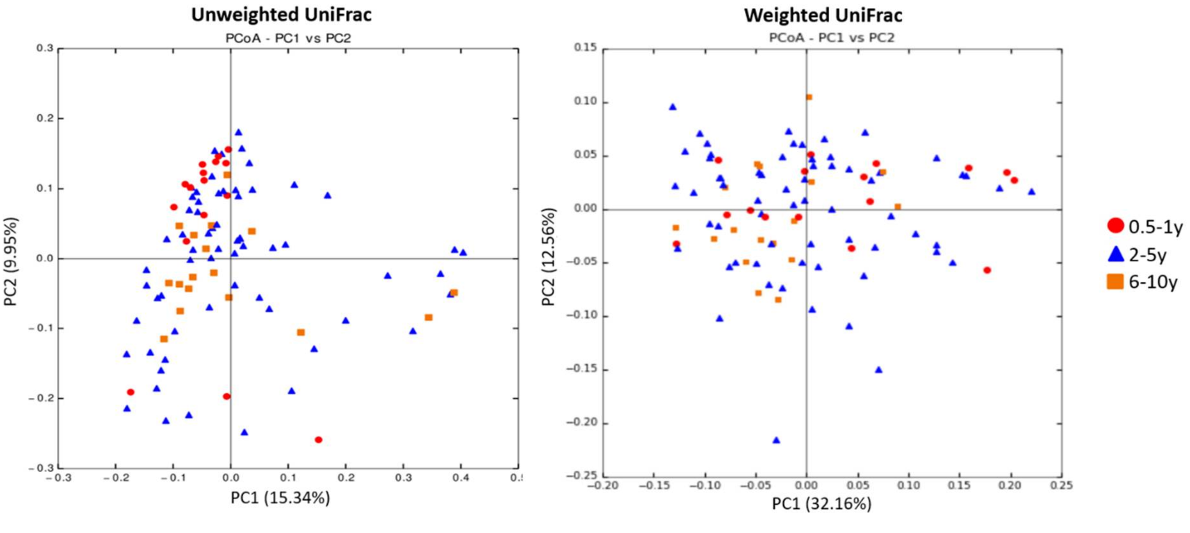

Supplementary Table S3. Relative abundance at the phylum and species level according to age. Kruskal–Wallis test was used, and data are shown as the mean ± SEM. Major or significantly different bacterial groups (abundance ≥ 0.1% or $P < 0.05$) are shown. Asterisks indicate $P < 0.05$.

	**0.5-1y**	**2-5y**	**6-10y**	**p-value**	**FDR**
**Phylum**					
Fusobacteria	9.9±2	14.5±1.3	17.6±2.2	4.5E-02*	3.2E-01
Tenericutes	0.3±0.1	0.4±0.1	0.5±0.1	1.5E-01	5.3E-01
Actinobacteria	5.7±2.6	3.4±0.7	1.7±0.6	3.4E-01	6.5E-01
Bacteroidetes	24.7±3.3	27.9±1.7	29.8±1.7	4.4E-01	6.5E-01
Deferribacteres	0.8±0.4	0.6±0.2	0.9±0.3	4.6E-01	6.5E-01
Firmicutes	49.6±5.6	44.2±2	42.6±2.9	6.5E-01	7.6E-01
Proteobacteria	9.2±2.1	9.2±1.1	7.1±0.7	9.6E-01	9.6E-01
**Genus**					
Jeotgalicoccus	0.1±0.1	0.2±0.1	0.1±0.1	1.9E-02*	6.1E-01
Faecalibaculum	0.4±0.2	0.5±0.2	0.1±0.1	2.7E-02*	6.1E-01
Fusobacterium	9.9±2.0	14.4±1.3	17.6±2.2	4.4E-02*	6.1E-01
**Species**					
Corynebacterium mastitidis	0.2±0.1	0.1±0.1	0.0±0.0	4.2E-04*	3.9E-02*
Corynebacterium ammoniagenes	0.0±0.0	0.0±0.3	0.1±0.1	2.7E-03*	1.3E-01
Clostridium oryzae	0.2±0.2	0.2±0.1	0.1±0.1	1.7E-02*	3.6E-01
Fusobacterium perfoetens	7.2±1.8	1.8±11.4	14.3±2.1	1.8E-02*	3.6E-01
Jeotgalicoccus nanhaiensis	0.1±0.1	0.1±0.2	0.1±0.1	1.9E-02*	3.6E-01
Faecalibaculum rodentium	0.4±0.2	0.2±0.5	0.1±0.1	2.7E-02*	4.3E-01
Clostridium saudiense	0.4±0.2	0.2±1.4	1.9±1.0	4.9E-02*	5.6E-01

Supplementary Figure S4: Linear discriminant analysis effect size (LEfSe) according to age. Cladogram showing different abundant taxa according to age (P < 0.05, LDA scores ≥ 3) and bar graph showing LDA scores.

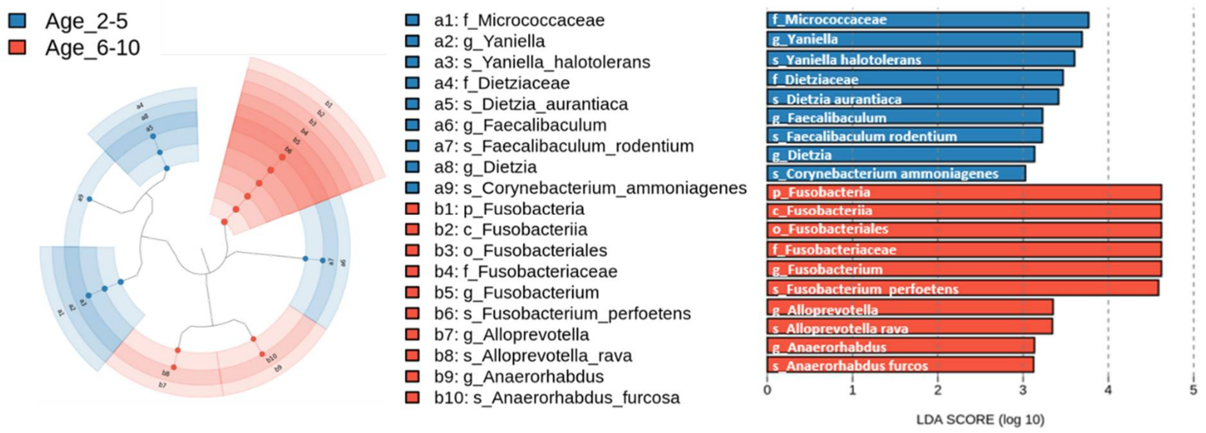

Supplementary Figure S5: Alpha and beta diversity according to body condition scores (BCS). (a) Microbial richness and evenness were measured based on observed OTUs and Shannon in dex, respectively. List of alpha diversity indices. (b) PCoA was performed based on un-weight ed and weighted UniFrac distances.

(a)

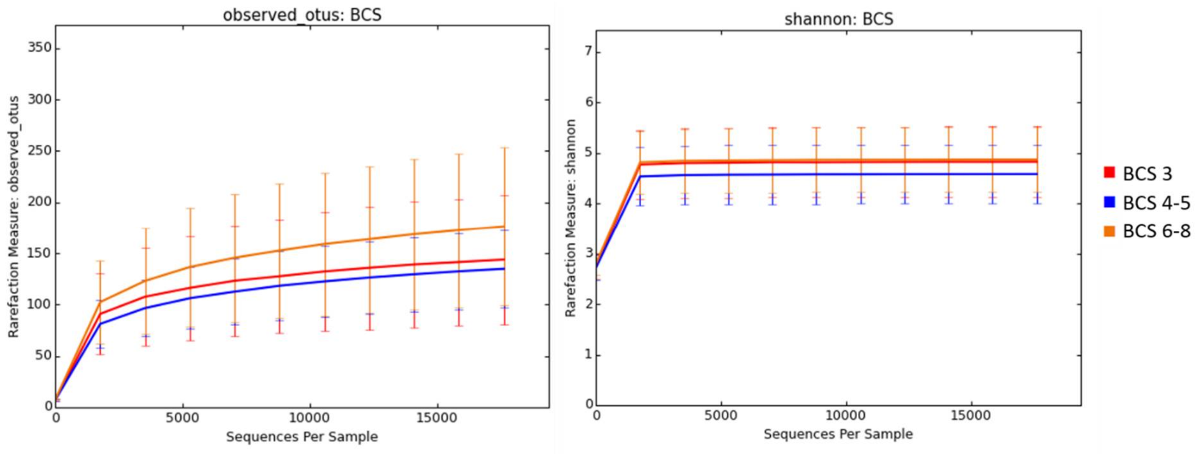

Diversity Index	BCS 3		BCS 4-5		BCS 6-8		p-value	FDR
	Mean	SEM	Mean	SEM	Mean	SEM		
Chao1	173.1	28.8	166.0	5.4	221.0	32.3	3.4E-01	6.4E-01
Inverse Simpson	0.9	0.0	0.9	0.0	0.9	0.0	6.4E-01	6.4E-01
Shannon	4.8	0.3	4.6	0.1	4.9	0.2	5.3E-01	6.4E-01
OTUs	160.3	28.5	149.5	4.7	200.2	29.1	3.3E-01	6.4E-01

(b)

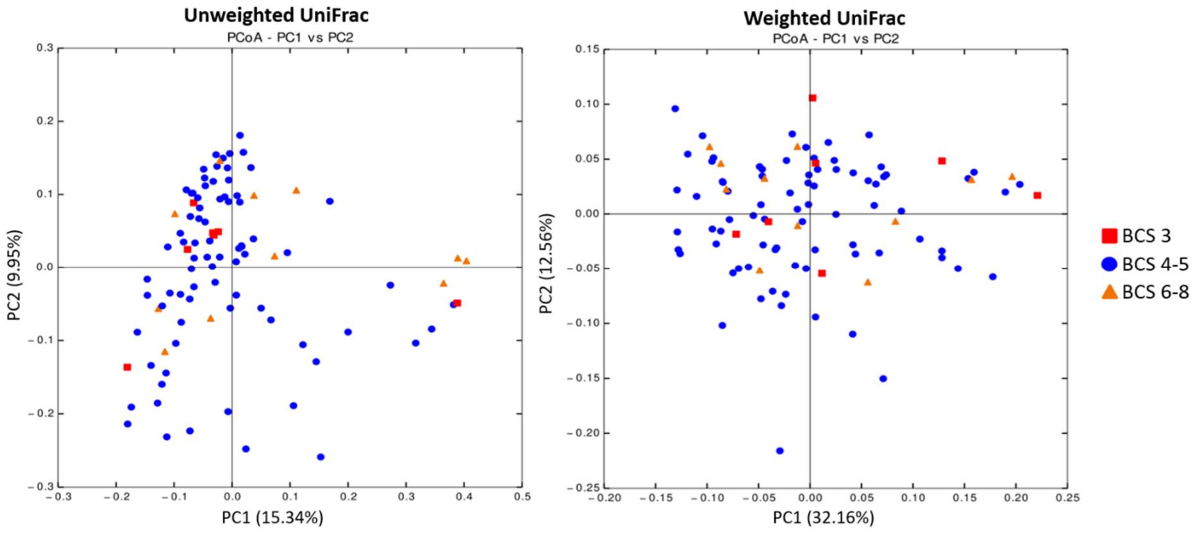

Supplementary Table S4. Relative abundance at the phylum and species level according to BCS. Kruskal–Wallis test was used, and data are shown as the mean ± SEM. Major or significantly different bacterial groups (abundance ≥ 0.1% or $P < 0.05$) are shown. Asterisks indicate $P < 0.05$.

	**BCS 3**	**BCS 4-5**	**BCS 6-8**	**p-value**	**FDR**
**Phylum**					
Actinobacteria	2.7±1.3	3.2±0.8	5.8±1.9	2.8E-02*	2.0E-01
Deferribacteres	0.8±0.5	0.8±0.2	0.2±0.1	1.3E-01	4.0E-01
Proteobacteria	6.1±1.1	9±0.8	9.3±3.5	2.2E-01	4.0E-01
Firmicutes	54.3±6.2	43.9±1.8	45.1±6	2.3E-01	4.0E-01
Fusobacteria	11.3±3.1	15±1.2	11.2±2	4.6E-01	5.5E-01
Tenericutes	0.3±0.1	0.4±0.1	0.3±0.1	4.7E-01	5.5E-01
Bacteroidetes	24.7±3.7	27.9±1.4	28.3±4.2	9.2E-01	9.2E-01
**Genus**					
Peptostreptococcus	0.2±0.2	0.1±0.1	0.2±0.2	1.9E-02*	7.7E-01
Schaalia	0.1±0.1	0.1±0.1	0.2±0.1	3.0E-02*	7.7E-01
Enterococcus	0.1±0.1	0.2±0.1	0.3±0.2	6.9E-02	7.7E-01
Streptococcus	1.3±0.7	1±0.4	0.5±0.4	7.2E-02	7.7E-01
Lactobacillus	11.7±4.4	7.7±1.1	9.7±3.2	2.4E-01	7.7E-01
**Species**					
Schaalia cardiffensis	0.1±0.1	0.1±0.1	0.2±0.1	8.2E-03*	7.9E-01
Peptostreptococcus russellii	0.2±0.2	0.1±0.1	0.2±0.1	1.8E-02*	7.9E-01
Enterococcus cecorum	0.1±0.1	0.1±0.1	0.2±0.1	2.7E-02*	7.9E-01
Romboutsia timonensis	11.3±3.1	6.2±0.9	4.1±0.9	1.6E-01	7.9E-01
Lactobacillus animalis	9.1±3.9	4.1±0.7	2.6±0.9	1.7E-01	7.9E-01
Lactobacillus johnsonii	0.3±0.1	1.0±0.3	2.6±1.4	2.4E-01	7.9E-01
Lactobacillus reuteri	0.8±0.4	1.0±0.2	1.4±0.5	2.9E-01	7.9E-01
Fusobacterium perfoetens	8.6±2.8	11.8±1.1	8.1±1.7	3.7E-01	8.8E-01
Prevotella copri	3.9±0.9	6.4±0.8	9.3±3.0	5.2E-01	9.1E-01

Supplementary Figure S6: Linear discriminant analysis effect size (LEfSe) according to BCS. Cladogram showing different abundant taxa according to BCS (*P* < 0.05, LDA scores ≥ 2) and bar graph showing LDA scores.

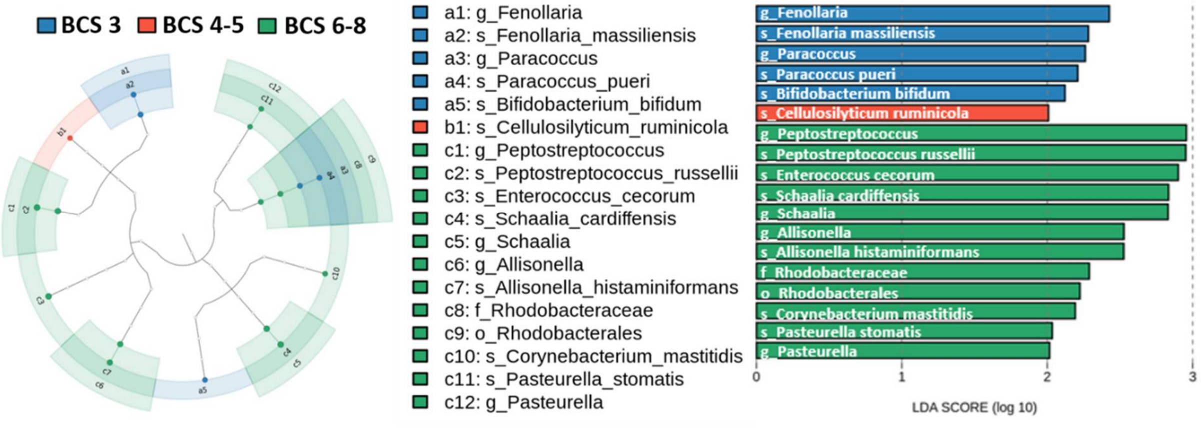

Supplementary Figure S7: Venn diagrams indicating the number of unique and shared operational taxonomic unit (OTU). OTUs detected across all samples were included in the analysis.

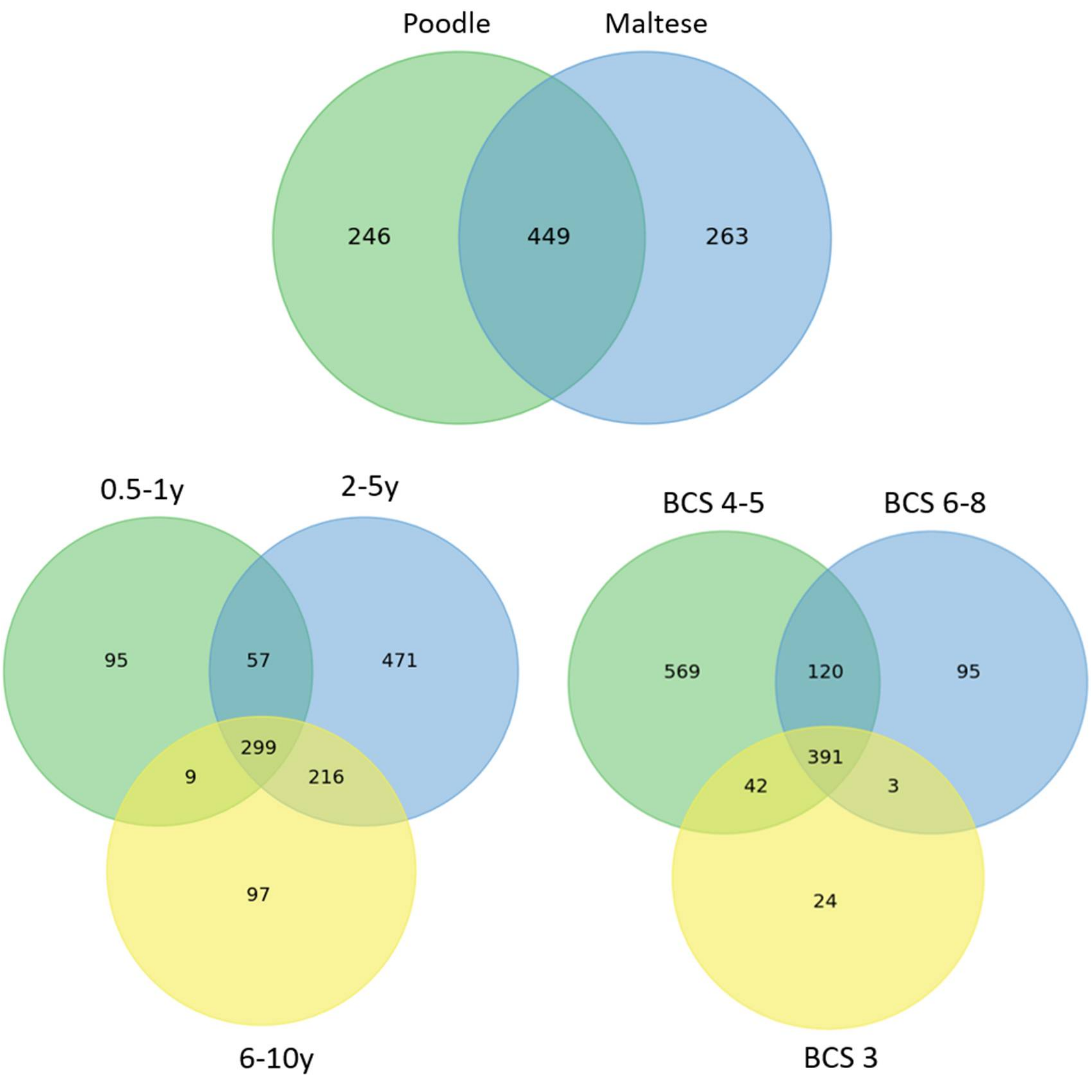

Supplement: Supplementary file 1 [file animals-11-02432-s001.zip › animals-1304517-supplementary.pdf]
